# Traditional Neurosyphilis in 21st Century – Tabes Dorsalis, Dementia Paralytica, Aseptic Meningitis and Unilateral Oculomotor Nerve Palsy in an HIV-Negative Man

**DOI:** 10.7759/cureus.18869

**Published:** 2021-10-18

**Authors:** Luis Landeiro, Renato Oliveira, Joana Graça, Raquel Gouveia

**Affiliations:** 1 Internal Medicine, Hospital da Luz Lisboa, Lisboa, PRT; 2 Neurology, Hospital da Luz Lisboa, Lisboa, PRT; 3 Neuroradiology, Hospital da Luz Lisboa, Lisboa, PRT

**Keywords:** immunocompetent, left oculomotor, dementia paralytica, tabes dorsalis, neurosyphilis

## Abstract

Syphilis is potentially a multisystem chronic infection caused by *Treponema pallidum*. Late symptomatic neurosyphilis has been less reported in developed countries, most often seen in untreated patients or in patients with HIV coinfection. We present a case of complicated neurosyphilis with widespread neurological involvement (dementia paralytica, tabes dorsalis, leptomeningitis and left oculomotor nerve involvement) presenting in the 21st century in an urban area of a well-developed European country in an HIV-negative patient.

## Introduction

Syphilis is potentially a multisystem chronic infection caused by *Treponema pallidum*. It is a sexually or vertically transmitted infection (STI) that, when left untreated, usually follows a disease course that is divided into primary, secondary, latent and tertiary disease. Tertiary syphilis develops in 15-40% of untreated patients, and can manifest with cardiovascular and neurologic complications, as well as severe skin, bony and visceral (gummas) lesions [1,2].

Neurosyphilis can occur at any stage of the infection, most often in untreated patients being twice as frequent in Human Immunodeficiency Virus (HIV) co-infected patients [3]. Neurosyphilis is uncommon nowadays, comparing with the era before the introduction of penicillin. Rates of neurosyphilis have been variable, ranging between 0.47 and 2.1 cases per 100,000 [3]. The United States Centers for Disease Control and Prevention (CDC) reports neurosyphilis as 0.8% of all syphilis cases [4].

In the early stages of the infection, frequent manifestations are asymptomatic or symptomatic meningitis, gumma and meningovascular syphilis. Late symptomatic neurosyphilis (dementia paralytica and tabes dorsalis) occurs in 10 to 20% of all untreated cases developing decades after the primary infection [3]. Ocular and otologic syphilis can occur at any time, often in the setting of acute meningitis [5]. Cranial nerve involvement is usually associated with meningeal disease, more often and in descending order of frequency: VII, VIII, VI and II [6].

We present a case of neurosyphilis with widespread neurological involvement presenting in the 21st century in an urban area of a well-developed European country in an HIV-negative patient.

## Case presentation

A 55-year-old single male was admitted for evaluation of progressive paraparesis, upper and lower extremities paresthesia (initially distal, with proximal progression), ataxic gait and left upper eyelid ptosis, progressing over four weeks. In the previous three to four months he had also experienced persistent low-intensity abdominal pain in the lower quadrants without irradiation or other gastrointestinal symptoms, as well as paroxysms of excruciating pain in both legs, most often at night.

The patient mentioned an episode of self-limited diplopia one year before the current admission, which lasted for a month and was evaluated through a brain MRI that was unremarkable.

There was no relevant prior medical history. The patient had no known allergies or sexual risk factors. Neurologic examination at admission revealed incomplete left eye ptosis, bilateral small pupils with sluggish light reflex but preserved accommodation, symmetric paraparesis (grade 4/5 on MRC scale); lower limbs areflexia; positive Romberg’s sign; lower limbs sensory ataxia, and hesitant, wide stand gait.

The remaining physical examination, as well as the routine laboratory parameters and brain CT scan, was unremarkable.

Cerebrospinal fluid (CSF) analysis demonstrated albuminocytologic dissociation (<5 cells, proteins 100mg/dL); glucose 59mg/dL (normal range 40-70mg/dL).

**Figure 1:**
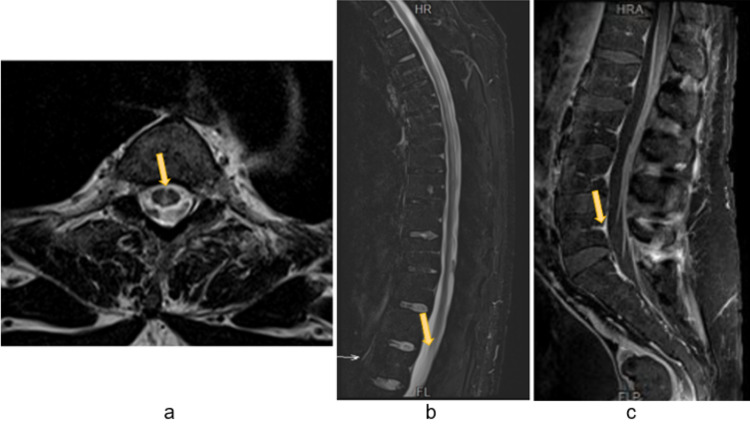
Spinal cord MRI - longitudinal T2-weighted hyperintensities in the dorsal columns of the spinal cord (arrows in panels a and b). Sagittal T1-weighted image showing pial conus medullaris and cauda equina gadolinium enhancement (arrow in panel c).

**Figure 2:**
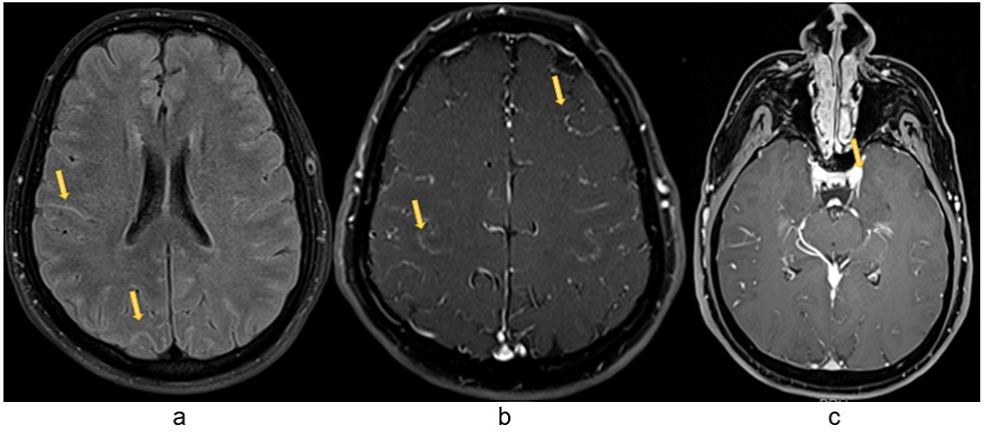
Brain MRI (FLAIR sequence) showing hyperintense signal in CSF space, especially in the sulci (arrows in panel a), diffuse pial signal gadolinium enhancement (arrows in panel b) as well as enhancement of both third cranial nerves, more intense on the left one (arrow in panel c). CSF: Cerebrospinal fluid; FLAIR: Fluid-attenuated inversion recovery.

Brain and spine MRI showed diffuse pial cerebral signal enhancement after gadolinium administration, as well as from both third cranial nerves, more intense on the left one; pial conus medullaris and cauda equina gadolinium enhancement; longitudinal T2-weighted hyperintensities in the dorsal columns of the spinal cord from C3 to D12 (Figures 1, 2).

As he was admitted because of a rapidly progressive ascending paraparesis with cranial nerve involvement, the hypothesis of Miller-Fischer acute demyelinating polyneuropathy was firstly assumed and treatment initiated with intravenous immunoglobulin - 0.4 mg/kg/24h.

Electromyogram was unremarkable, performed three days after admission (approximately one month after symptom onset). Chest-abdomen-pelvis CT did not show any evidence of occult tumor.

The Venereal Disease Research Laboratory (VDRL) test was found to be positive with a 1/16 titer in CSF and 1/32 titer in serum. Serum Treponema pallidum hemagglutination (TPHA) test was positive with a titer of 1/2560.

The remaining CSF and serum serologies were negative - Brucella, Borrelia burgdorferi, Campylobacter jejuni, Leptospira, Listeria monocytogenes, Mycoplasma pneumoniae, Herpes simplex, Varicella zoster and Human T-lymphocyte virus infection. Tuberculosis was also excluded. HIV 1 and 2 were not detected. Additionally, antiganglioside antibodies, thyroid-stimulating hormone, angiotensin-converting enzyme, vitamin B12 and folate were not detected or within normal range.

Echocardiography and carotid doppler ultrasound showed no evidence of cardiovascular involvement. Ocular syphilis was also excluded through observation by a neuro-ophthalmologist.

The patient was treated with intravenous penicillin 24 million units per day for 14 days. Up until discharge, one could see behavioral changes not so obvious at admission, such as marked anxiety and mild paranoia. Through the patient medical records, we were later able to find a neuropsychological evaluation (including multiple test batteries) from five years before the current admission, that showed mild cognitive impairment, psychic retardation, inability to learn and to manage complex information.

The patient had a mild improvement at discharge, being able to walk without aid, maintaining a wide-based gait. The pain paroxysms resolved but dysesthesias and paresthesia persisted.

Clinical follow-up up until the 18th month revealed persistent gait ataxia and left eye ptosis and recovery of muscular strength. Lower limb pain persisted, mildly controlled with gabapentin, titrated up to 2000mg per day. Neuropsychiatric symptoms (insomnia, anxiety, depressive mood and mild paranoia) were moderately improved with quetiapine 50mg and escitalopram 20mg once a day. CSF analysis six and twelve months after treatment showed persistent pleocytosis (40% lower than the first measurement, approximately 60mg/dL), as well as an 8- and 16-fold decrease in CSF-VDRL titer, respectively.

Brain MRI was repeated at six and 12 months after treatment, being similar to the initial one. Brain MRI at 18 months after treatment showed slightly less intense signal enhancement after gadolinium administration in the spinal cord, particularly in the medullary cone.

Serum VDRL titer was only halved at the 18th month after treatment. The patient refused further lumbar punctures.

## Discussion

Neurosyphilis was common in the pre-antibiotic era, occurring in 25 to 35 percent of patients with syphilis. One-third had asymptomatic neurosyphilis and the remaining had one of the symptomatic disease types. Nowadays it is an unusual diagnosis, mostly seen in its early forms and in HIV coinfected patients or in low-developed countries [2,7].

We present the case of an HIV-negative 55-year-old male, in whom a complex form of neurosyphilis was diagnosed. He had clinical and complementary diagnostic test results compatible with dementia paralytica (mild cognitive impairment and psychiatric symptoms), tabes dorsalis (sensory ataxia, ataxic gait, symmetric paraparesis, lower limbs areflexia, Argyll Robertson pupils, spine MRI with posterior roots uptake) and third cranial nerve involvement (left eyelid ptosis and third cranial nerve enhancement after gadolinium administration) [3].

The MRI changes suggested a diffuse inflammatory demyelinating disease that supported the initial diagnostic option of Miller Fisher Syndrome, which can involve the posterior cords, cauda equina roots and cranial nerves [8,9]. This hypothesis was cast aside when neurosyphilis was diagnosed, as the latter explains all clinical and radiological features and it would be extremely unusual to have both diseases simultaneously. Subacute combined degeneration was a less likely hypothesis due to the leptomeningeal and radicular involvement [10]. All other infectious, autoimmune and paraneoplastic diagnostic hypotheses were excluded through imaging results and extensive CSF and serum analysis.

CSF analysis was also unusual, as it presented with albuminocytologic dissociation. The average number of CSF cells in patients with tabes dorsalis and general paresis is 25-75 cells/mL, but in 10% of those with tabes dorsalis the CSF cell count may be normal (often called “burned out stage” of the disease), which is what happened in our patient [7,11].

Third cranial nerve involvement, as evidenced by left eyelid ptosis and its enhancement after gadolinium administration, is a rare feature of neurosyphilis (seventh, eighth, sixth and second cranial nerves are far more commonly involved), usually associated with the meningeal forms of the disease [6].

Additionally, one should try to integrate the episode of diplopia from the previous year in the current diagnosis, as it could already have been a syphilis clinical feature, such as syphilitic meningitis or meningovascular disease with cranial nerve palsy. It was not possible to gather any more information regarding the clinical features of this episode of diplopia as well as the MRI report or images. It is difficult to assess which of them really occurred, but assuming the absence of meningeal signs, fever, hypoglycorrhachia and an apparent long course of infection favors the latter.

Regarding follow-up, even though we saw a 16-fold reduction in CSF-VDRL titer, we have seen mild improvements in MRI findings or in clinical symptoms, which is in line with what may be expected in the late parenchymatous forms of neurosyphilis, as one hopes to halt disease progression but it is rarely seen significant clinical improvement [3,7].

Therefore, we present a complex and atypical case of late neurosyphilis, with clinical features of dementia paralytica, tabes dorsalis, leptomeningitis and left oculomotor nerve involvement, with only a partial clinical response to treatment, despite the serological proof of cure.

## Conclusions

Although less common, late forms of neurosyphilis must always be searched for in cases where there is the slightest suspicion, since this is a disease with very complex and heterogeneous presentations as well as a specific treatment with prognostic impact. We present a complex and atypical case of late neurosyphilis, with clinical features of dementia paralytica, tabes dorsalis, leptomeningitis and left oculomotor nerve involvement. Its partial clinical and radiological response to treatment, despite the serological proof of cure, is often seen in the late parenchymatous forms of neurosyphilis, which bolsters the need of early diagnosis.
